# A new species of the subgenus Scymnus from Pakistan (Coleoptera, Coccinellidae)

**DOI:** 10.3897/zookeys.694.12863

**Published:** 2017-08-29

**Authors:** Azad Rashid, Xiaosheng Chen, Baoli Qiu, Xingmin Wang

**Affiliations:** 1 Key Laboratory of Bio-Pesticide Innovation and Application, Engineering Technology Research Center of Agricultural Pest Biocontrol, Guangdong Province; Department of Entomology, South China Agricultural University, Guangzhou 510640, China; 2 Department of Forestry Protection, College of Forestry and Landscape Architecture, South China Agricultural University, Guangzhou, 510640, China

**Keywords:** Coccinelloidea, Coccidulini, entomology, new species, Pakistan, taxonomy

## Abstract

A new species, Scymnus (Scymnus) contortubus Rashid, Chen & Wang, **sp. n.**, is described and illustrated from Pakistan. A diagnosis, remarks, illustrations, and a distribution map are provided of the new species and its most similar congener, S. (S..) nubilus Mulsant.

## Introduction

The subgenus Scymnus was established by Mulsant, 1850 with *Coccinella rufipes* Fabricius, 1798 as the type species, based on the presence of an 11-segmented antenna and the incomplete abdominal postcoxal line.

The genus *Scymnus* Kugelann, 1794 belonged to the tribe Scymnini Mulsant, 1846 in the subfamily Scymninae (Sasaji 1968; Kovář 1996). However, Ślipiński (2007) presented a two-subfamily system, moving the genus *Scymnus* to the tribe Coccidulini in the subfamily Coccinellinae. Further studies based upon molecular and morphological characters supported Ślipiński’s (2007) division of Coccinellidae in two subfamilies, Coccinellinae and Microweiseinae (Giorgi et al. 2009; Seago et al. 2011). Recent study on the Cucujoidea (Robertson et al. 2015) has recovered Coccinellidae as belonging to the superfamily Coccinelloidea together with eight other families of the former Cerylonid Series.

Members of the genus *Scymnus* Kugelann, 1794 are predatory and mostly feed on aphids, adelgids, and scale insects, playing an important role in regulating pest populations (Chen et al. 2016). Prior to the present study, 58 species were reported in the subgenus Scymnus (Scymnus) from the Oriental Region and only one species, S. (S..) nubilus Mulsant, 1850, has been known to exist in Pakistan (Poorani, 2002; Chen et al. 2013). In this paper, another species is described, and a diagnosis of the subgenus with distribution pattern of both species is presented.

## Materials and methods

Specimens were collected from different localities in Pakistan during 2015–2016 and were preserved in 85% ethanol. A Zeiss Stemi 305 microscope was used for observing external morphology followed by the dissection of male and female genital structures. After dissection, genitalia were cleared in 10% solution of NaOH and placed in a drop of neutral balsam onto glass slides for further studies.

SteREO Discovery V20 (Zeiss) microscope with an ocular micrometer was used for all measurements, which are presented in millimetres. The following abbreviations are used:

**TL** total length from clypeus to apex of elytra,

**TW** total width across both elytra at widest part,

**TH** total height in highest part of elytra,

**HW** head width in widest part including eyes,

**PL** pronotal length across the central area from anterior to basal margin of pronotum,

**PW** pronotal width across widest part,

**EL** elytral length along suture including scutellum,

**EW** elytral width, equivalent to TW.

An AxioCam HRc digital camera attached to the stereoscope, (SteREO Discovery V20) was used for photographs of the whole bodies of beetles. Composite images were generated with AXIO VISION REL. 4.8 software and edited using ADOBE PHOTOSHOP CC. 2017.

A compound microscope, Olympus BX51 attached to a Coolsnap-Procf & CRI Micro*Color camera was used for the preparation of illustrations of the morphological characters of male genitalia. Morphological terminology of Ślipiński (2007) was followed. Type specimens are deposited in the Department of Entomology, South China Agricultural University, Guangzhou, China.

## Taxonomy

### Genus *Scymnus* Kugelann, 1794

#### Subgenus Scymnus

Taxon classificationAnimaliaColeopteraCoccinellidae

Kugelann, 1794

Scymnus Kugelann, 1794: 545. Type species: designated by Westwood 1838: 43, Scymnus nigrinus Kugelann, 1794.
Anisoscymnus Crotch, 1874: 273. Type species: Coccinella rufipes Fabricius, 1798, by original designation.

##### Diagnosis.

**Figure 1.**
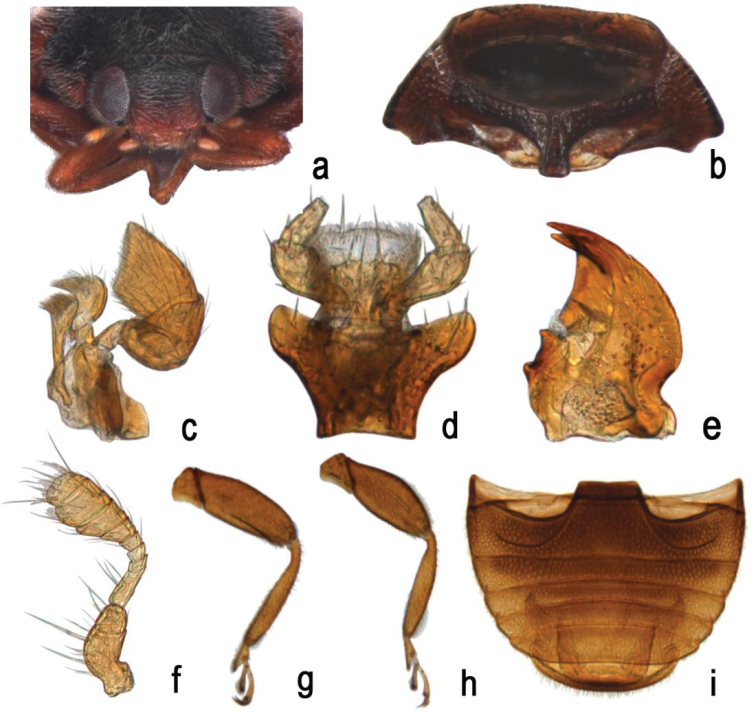
Scymnus (Scymnus) contortubus Rashid, Chen & Wang, sp. n.: **a** head, frontal **b** prothorax, antero-ventral **c** maxilla **d** labium **e** mandible **f** antenna **g** fore leg **h** hind leg **i** abdomen, male.

Members of the subgenus Scymnus can be easily distinguished by the following combination of characters: body small, oval or elongate oval; antennae 11-segmented (Fig. 1f); prosternum having distinct carinae, nearly reaching the anterior margin (Fig. 1b); abdominal postcoxal line incomplete, recurved forward, never reaching lateral margin of ventrite; area surrounding by postcoxal line sparsely punctate; abdomen with six ventrites, 5^th^ and 6^th^ abdominal ventrites in male truncate or emarginate apically in male (Fig. 1i); female genitalia with distinct infundibulum.

##### Distribution.

Worldwide (Chen et al. 2013).

#### Scymnus (Scymnus) contortubus

Taxon classificationAnimaliaColeopteraCoccinellidae

Rashid, Chen & Wang sp. n.

http://zoobank.org/C6D02733-EB0F-4EC9-964D-4F6FABBCC6C2

Figs 1 , 2

##### Etymology.

The species name is derived from Latin (*contortum* = twisted and *tubus* = tube) referring to a curved, tube-like apex of penis.

##### Diagnosis.

**Figure 2.**
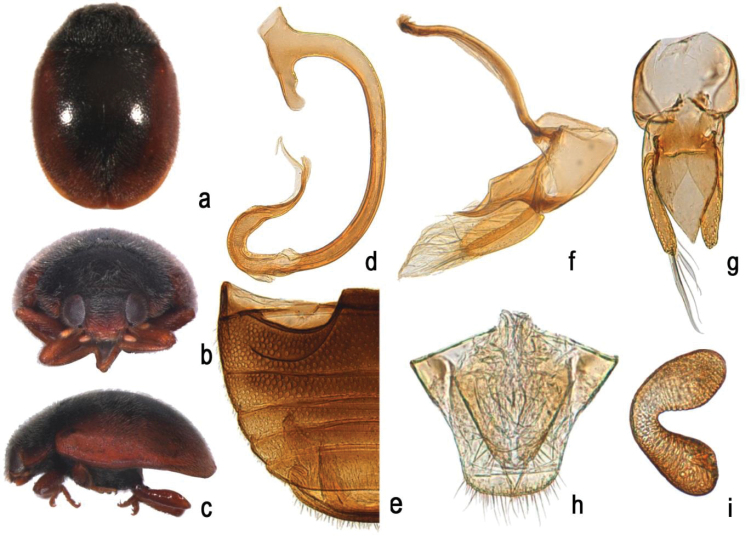
Scymnus (Scymnus) contortubus Rashid, Chen & Wang, sp. n.: **a** dorsal view **b** anterior view **c** lateral view **d** penis **e** abdomen **f** tegmen, lateral view **g** tegmen, ventral view **h** ovipositor **i** spermatheca.

**Figure 3.**
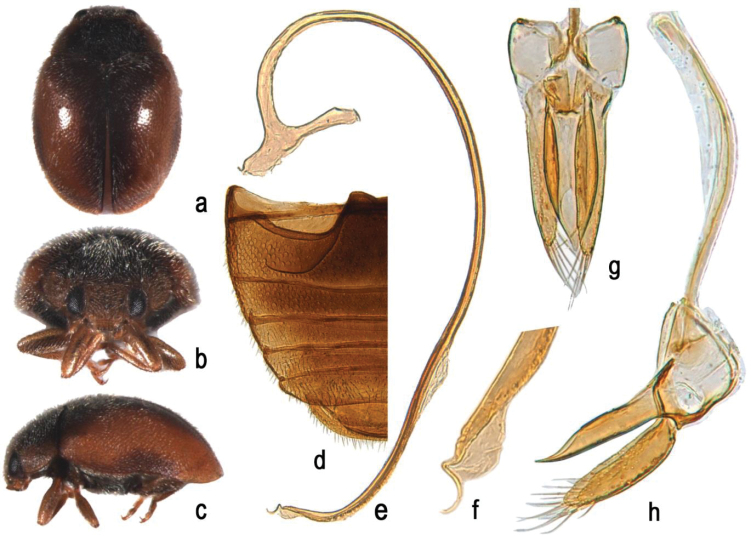
Scymnus (Scymnus) nubilus Mulsant: **a** dorsal view **b** anterior view **c** lateral view **d** abdomen **e** penis **f** apex of penis **g** tegmen, ventral view **h** tegmen, lateral view.

This species is separated by the presence of a stout penis with a curved apex (Fig. 2d) and parameres with long, dense setae on both the apex and inner side (Fig. 2f–g). In S. (S..) nubilus, the most similar congener, penis is slender (Fig. 3e) and parameres have sparse long setae only at apices (Fig. 3g–h).

##### Description.

TL: 1.8 mm, TW: 1.3 mm, TH: 0.7 mm, HW: 0.6 mm, TL/TW: 1.38, PL/PW: 0.33, EL/ EW: 1.07.

Body oval, distinctly convex, dorsum with dense white pubescence. Head brown with black vertex. Antennae and mouthparts brown. Pronotum black with lateral margins reddish brown. Elytra reddish brown with broad black U-shaped sutural stripe, extending 5/6 length of elytral suture (Fig. 2a–c). Lateral margins of elytra coarsely punctate. Prothoracic hypomeron reddish brown. Prosternum, mesoventrite, and metaventrite black. Elytral epipleuron yellowish brown with both margins black. Legs reddish brown.

Head small, width 0.66 times of pronotal width (HW/PW= 0.6/0.9). Head with fine punctures, same size as eye facets, separated by 0.5–1.0 diameters (Fig. 2b). Eyes densely faceted, with dense hairs, interocular distance 0.47 times head width. Pronotum width 0.69 times of elytral width (PW/EW=0.9/1.3), pronotal punctures sparse, slightly coarser than those on head, 1.0–2.0 diameters apart. Elytral punctures coarse, separated by 2.0–4.0 diameters. Prosternal carinae distinct extending to anterior margin and slightly converging anteriorly. Prosternal process T-shaped, twice as long as its width at base. Abdominal postcoxal lines incomplete, extending to 6/7 length of ventrite and slightly recurved toward base of ventrite; area surrounding postcoxal line with dense granular punctures, distributed unevenly (Fig. 2e).

*Male genitalia*: Penis stout (Fig. 2d). Penis capsule with long inner process and short dilated outer one. Apex of penis strongly recurved towards inner side with thread-like appendage. Tegmen stout, with penis guide parallel-sided from base to nearly half length, tapering gradually to a blunt apex in ventral view. In lateral view, penis guide with sides parallel at basal 2/3, then abruptly constricted to pointed apex. Parameres stout; slightly longer than penis guide, with dense, long setae on apical and inner margins (Fig. 2f–g).

*Female genitalia*: Coxites elongate triangular, 2.5 times as long as wide, tapering to blunt apices, each with long terminal setae (Fig. 2h); infundibulum present; spermatheca C-shaped (Fig. 2i).

##### Types.

Holotype, Male, **PAKISTAN**: (Kashmir). Arja Mountains, No. SCAU (E) 16579, [N33°57 27.80, E073°39 54.04"], *ca.* 940 m, 28.X.2015, Huo LZ leg; Paratypes: (86) 2♂1♀, same data as holotype; 8♂1♀ Rawalakot, [N33°52 07.93", E073°43 46.49"] *ca.* 1668 m; 28.X.2015, Huo LZ leg; 12♂ 10♀ Mirpur, [N33°28 23.07", E073°52 57.94"] *ca.* 500–610 m, 26.X.2015; Huo LZ leg; (Khyber Pakhtunkhwa) 5♂ 5♀, Balakot, [N34°33 27.62", E073°21 25.08"] *ca.* 1093 m; 15.X.2015; Wang XM leg; 1♂ Birote, [N34°03 27.54", E073°30 00.32"] *ca.* 789 m; 13.X.2015; Wang XM leg; 4♂ 2♀ Arab khan, [N34°25 20.73", E073°18 44.21"] *ca.* 104 m; 16.X.2015; Wang XM leg; 5♂ 6♀ Shung, [N34°52 31.52", E072°54 12.46"] *ca.* 656 m; 17.X.2015; Wang XM leg; 9♂ ♀7 Parehna, [N34°21 32.17", E073°04 53.75"] *ca.* 775 m; 19.X.2015; Wang XM leg; 1♂ 1♀ Paras, [N34°39 23.53", E073°30 13.91"] *ca.* 1364 m; 15.X.2015; Wang XM leg; (Punjab) 1♂ 2♀ Salgran, [N33°49, 33.96", E073°17 09.48"] *ca.* 857 m; 13.X.2015; Wang XM leg; 2♂ 1♀ Gokina [N33°46, 03.26", E073°04 37.78"] *ca.* 1090 m; 11.X.2015; Wang XM leg.

##### Distribution.

Pakistan (Kashmir, Khyber Pakhtunkhwa, Punjab).

#### Scymnus (Scymnus) nubilus

Taxon classificationAnimaliaColeopteraCoccinellidae

Mulsant, 1850

Fig. 3

Scymnus nubilus Mulsant, 1850: 972; Bielawski 1972: 293; Booth and Pope 1989: 359; Canepari 2001: 207; Chen et al. 2013: 438.
Scymnus (Scymnus) nubilus : Korschefsky 1931: 143; Kapur 1972: 311; Miyatake 1985: 9; Yu and Pang 1992: 44; Poorani 2002: 358; Kovář 2007: 590; Yu 2011: 131; Chen et al. 2013: 438.

##### Remarks.

Scymnus (S..) nubilus Mulsant can be easily confused with S. (S..) contortubus sp. n. and other *Scymnus* species due to their similar colouration. However it can be distinguished from other species by the long and slender penis with nearly S-shaped tip (Fig. 3e–f). Diagnostic are also: the tegmen stout (Fig. 3g–h), the penis guide nearly parallel along 2/3 of basal length, converging to pointed apex in ventral view (Fig. 3g), in lateral view abruptly narrowing from apical 1/3 length to apex; parameres with long apical setae (Fig. 3h).

##### Material examined.

**PAKISTAN** (Kashmir) 1♀ 2♂, Mirpur, [N33°28 23.07", E073°52 57.94"] *ca.* 500–610 m, 26.X.2015, Huo LZ leg. (Khyber Pakhtunkhwa) 1♀ 1♂, Besham [N34°59 51.61", E072°54 21.34"] *ca.* 750 m, 18.10.2015, Wang XM leg; 1♂ Arab khan, [N34°25 20.73", E073°18 44.21"] *ca.* 1043 m, 16.X.2015, Wang XM leg. (Punjab) 1♀, Chakwal, [N32°55 33.23", E072°44 56.91"] *ca.* 515 m, 20.X.2015, Wang XM leg; 3♂, Kallar kahar, [N32°46 20.29", E072°42 56.28"] *ca.* 648 m, 21.X.2015, Wang XM leg; 3♀ 1♂, Margalla [N33°44 00.16", E073°02 12.16"] *ca.* 609 m, 10.X.2015, Wang XM leg; 1♂, Pai khel [N32°45 57", E071°34 21"] *ca.* 226m , 17.IX.2016, Rashid A leg; 2♂, Khurianwala, [N31°31 41.54", E073°14 50.86"], *ca.* 192 m, 22.X.2015, Wang XM leg.

##### Distribution.

Pakistan (Kashmir, Khyber Pakhtunkhwa, Punjab), China, Japan, India, Sri Lanka, Burma, Nepal, Micronesia, Portugal, La Réunion, African Region.

**Figure 4.**
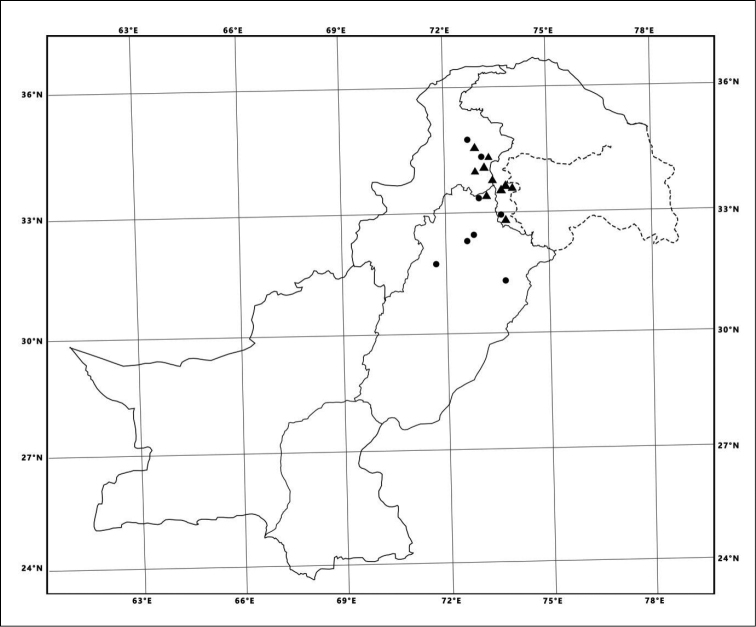
Distribution map: Scymnus (Scymnus) contortubus Rashid, Chen & Wang, sp. n. (▲) and Scymnus (Scymnus) nubilus Mulsant (●).

## Supplementary Material

XML Treatment for Scymnus

XML Treatment for Scymnus (Scymnus) contortubus

XML Treatment for Scymnus (Scymnus) nubilus
